# A multi-mode super-fano mechanism for enhanced third harmonic generation in silicon metasurfaces

**DOI:** 10.1038/s41377-023-01134-1

**Published:** 2023-04-21

**Authors:** David Hähnel, Christian Golla, Maximilian Albert, Thomas Zentgraf, Viktor Myroshnychenko, Jens Förstner, Cedrik Meier

**Affiliations:** 1 Theoretical Electrical Engineering & CeOPP, Paderborn University, 33098 Paderborn, Germany; 2 Physics Department & CeOPP, Paderborn University, 33098 Paderborn, Germany

**Keywords:** High-harmonic generation, Nanophotonics and plasmonics

## Abstract

We present strong enhancement of third harmonic generation in an amorphous silicon metasurface consisting of elliptical nano resonators. We show that this enhancement originates from a new type of multi-mode Fano mechanism. These ‘Super-Fano’ resonances are investigated numerically in great detail using full-wave simulations. The theoretically predicted behavior of the metasurface is experimentally verified by linear and nonlinear transmission spectroscopy. Moreover, quantitative nonlinear measurements are performed, in which an absolute conversion efficiency as high as *η*_max_ ≈ 2.8 × 10^−7^ a peak power intensity of 1.2 GW cm^−2^ is found. Compared to an unpatterned silicon film of the same thickness amplification factors of up to ~900 are demonstrated. Our results pave the way to exploiting a strong Fano-type multi-mode coupling in metasurfaces for high THG in potential applications.

## Abstract (web summary)

We theoretically and experimentally show a 900-fold boosting of optical THG emission in silicon metasurfaces using a novel type of nonlinear multi-mode Fano mechanism.

## Graphical abstract

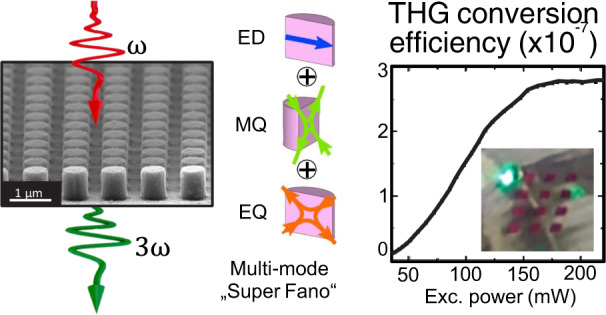

## Introduction

Nonlinear optics has been an active field of research over the past several decades providing us with the ground for many physical processes and phenomena such as high-harmonic generation, spontaneous parametric up/down conversion, stimulated Raman scattering, the non-linear Kerr effect, etc^1^. Such kind of processes form the essence of a wide range of applications which extends from ultrafast pulse characterization^2–4^ and frequency-resolved optical gating (FROG)^5,6^ to novel light sources^7–9^ and ultrafast nanoscale devices as optical sensors^10^. Recently, enhanced generation of nonlinear light on a subwavelength scale using metasurfaces has attracted booming attention due to their ultrathin compact profile and advanced functionalities relevant for modern integrated photonics^11–13^. In comparison to their bulk counterparts, nonlinear metasurfaces do not suffer from phase matching condition problems and can utilize the resonant nature of the nanoantennas they are formed of ref. ^14^. Initially, plasmonic metasurfaces, which support surface plasmon resonances^15^, have been extensively used for enhancing high-harmonic generation^16,17^. However, they typically possess only a relatively low nonlinear surface susceptibility, high absorption losses caused by ohmic dissipation in metals, and have low damage thresholds. As an alternative, dielectric metasurfaces have lately emerged offering many possibilities to significantly increase the light-matter interaction via tailoring optically induced Mie-type resonances hosted within nanoparticles made of high refractive index materials. The inherently rich variety of electric and magnetic multipolar resonances can be spectrally tailored by varying the size and shape of the nanoparticles, which can lead to their mutual overlap and even swap positions in the spectrum. This can give rise to the exotic phenomena, such as the excitation of Fano resonances^18–20^, nonradiative modes^21,22^, and resonances associated with bound states in the continuum (BIC)^23–26^, which possess ultra-high quality factors. High-quality modes supported by all-dielectric metasurfaces in combination with negligible ohmic losses compared to plasmonic devices, low heating, and high excitable mode volume have led to their emerging application for the efficient nonlinear light generation^27–29^.

In particular, the BIC concept is extensively exploited in the generation of enhanced third harmonic generation (THG) in metasurfaces composed of silicon nano resonators with the latest record peak power independent conversion efficiency of 3.9 × 10^−4^W^−2^ at a peak power intensity of 1 GW cm^−2^ reported by ref. ^26^. Also, in other metasurfaces consisting of materials with a very large nonlinear susceptibility component, such as germanium, gallium arsenide, aluminum gallium arsenide, or zinc oxide, a substantial enhancement of the harmonic generation processes (SHG and THG) has been demonstrated^30–33^. This development in the nonlinear metasurfaces opened up new ways to advance nonlinear wavefront design with a variety of exciting applications, such as nonlinear holographic imaging^34–37^, beam steering^38,39^, and generation of entangled photons by spontaneous parametric downconversion^13,40,41^.

In this paper, we report on theoretical and experimental demonstration of a completely new Fano-resonant all-dielectric metasurface possessing large THG. We utilize an amorphous silicon (a-Si) nanocylinder of an elliptical form as the building block of the nonlinear metasurface. By tailoring multipolar Mie resonances in these nano resonators via geometrical parameters, we achieve a THG enhancement up to ~900 compared to an unpatterned silicon film of the same thickness and a conversion efficiency of *η*_max_ ≈ 2.8 × 10^−7^ at a peak power intensity of 1.2 GW cm^−2^. Our extremely enhanced THG is generated by the interaction of an ultra-narrow high-Q quadrupolar magnetic mode with the two broad electric dipole and electric quadrupole modes, which are spectrally brought so close together that they form what we call the ‘Super-Fano’ resonance. Our full-wave simulations are in good qualitative agreement with the experimental results obtained from linear and nonlinear transmission spectroscopy on the fabricated metasurface.

## Results

### Numerical analysis

**Fig. 1:**
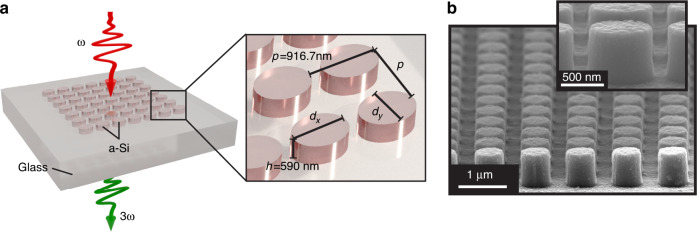
Investigated setup. **a** Schematic of the fabricated all-dielectric metasurface for enhanced THG along with design parameters. **b** SEM image of the fabricated Si antennas. A single antenna is seen in the inset (sample is carbon-coated to mitigate charging effects)

Our metasurface consists of a periodic arrangement of a-Si elliptical cylinders on a glass substrate, see Fig. 1a. We used a Monte Carlo method to sample over a large ensemble of parameters and determined a range of height and lattice constant parameters leading to enhanced THG^42^. We selected a lattice constant of *p* = 916.7 nm, which we kept constant in all our calculations to exclude any undesired influence from lattice effects. For the resonator height, we chose *h* = 590 nm, which is expected to be thick enough to sustain dielectric mode resonances even at the fundamental frequencies. The two remaining parameters *d*_*x*_ and *d*_*y*_ vary in a range from 350 nm to 850 nm.

**Fig. 2:**
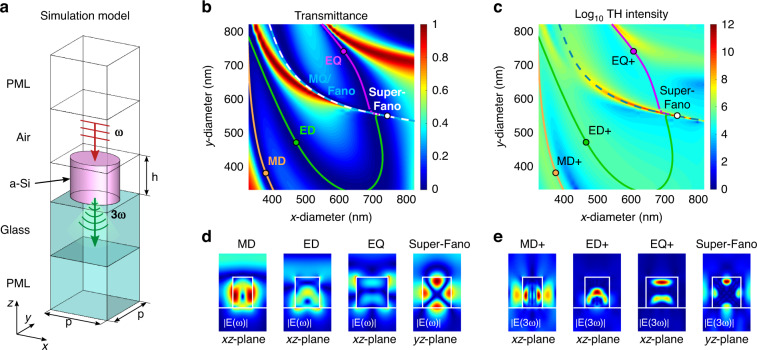
Numerical analysis. **a** Simulation model of a unit cell for the infinitely periodic metasurface consisting of a-Si resonators. **b** Calculated transmittance and **c** TH intensity (log scale) maps as a function of elliptical diameters *d*_*x*_ and *d*_*y*_ of the resonator. The colored curves plotted in both maps highlight the paths of the FF mode resonances (**b**). **d**, **e** Calculated electric near-field plots of modes for different resonators with diameters indicated by points in the maps at (**d**) pump 1560 nm and (e) TH 520 nm wavelengths. The periodicity of the unit cell and the resonator height are *p* = 916.7 nm and *h* = 590 nm, respectively. The maximum of the generated TH is indicated by the dot with label Super-Fano in both maps for the resonator with diameters *d*_*x*_ = 740 nm and *d*_*y*_ = 550 nm

We start our analysis by studying the linear behavior of the metasurface. The result of numerical calculations of the linear transmittance is shown in Fig. 2b. This map unveils the rich structure with multiple Mie-modes, suggesting their mutual interaction when they get within close vicinity inside the parameter space. It should be noted here that the modes in the map appear as dark-blue colored valley regions, since an optical mode causes absorption of light, i.e., a low transmission value. We identified at least four resonances indicated by the contour isolines (colored curves) with corresponding labels. Figure 2d shows the electric-field amplitude maps |*E*(*ω*)| associated with these resonances in the different planes at *y* = 0. From the symmetry characteristics of these fields revealing 2- and 4-fold symmetries, we can assign the four main valleys in the map to the broad magnetic (MD) and electric (ED) dipolar modes as well as the magnetic (MQ) and electric (EQ) quadrupolar modes.

**Fig. 3:**
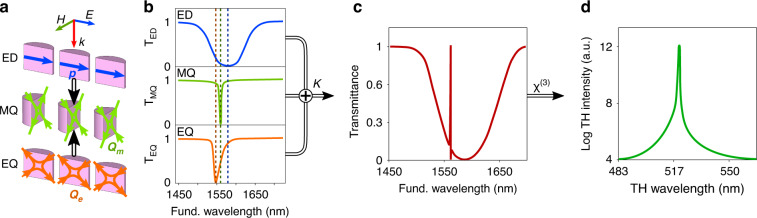
The multi-mode Fano resonance mechanism. **a** Illustration of the enhanced third harmonic generation scheme by the Super-Fano resonance mechanism. This formation involving electric dipole, magnetic quadrupole, and electric quadrupole modes. **b** Transmittance spectra associated with the corresponding modes forming single resonances. **c** The resultant transmittance spectrum originated from coupling of these three modes with strength *κ*, forming the Fano-like sharp asymmetric resonance profile. **d** The corresponding TH spectrum of high intensity

As a more detailed analysis of the resonances reveals (also see spectra in Supplementary Fig. S3), the narrow magnetic quadrupole mode interacts over a wide range of diameter parameters with the nearly parallel broad continuum electric dipole mode (green curve), forming a classical asymmetric Fano resonance feature indicated by the dashed curve and the MQ/Fano label in the transmittance map Fig. 2b. By adjusting the diameters of the resonator along this curve, the spectral width and thus the Q-factor and radiated THG of the Fano resonance can be easily optimized. Intriguingly, the sharpest Fano resonance feature and the strongest THG emission (approx. five orders of magnitude enhancement) is obtained at the point, we label as the Super-Fano resonance in Fig. 2b, where the MQ/Fano resonance comes in close contact with a third resonance, the electric quadrupole mode, indicated by the magenta curve. Unexpectedly, this point is slightly shifted from the supposed intersection of the three curves, ED, MQ/Fano and EQ, because the MQ mode acts in this case as a barrier for the broad ED and EQ resonances, resulting in a bending of the curves closely before the intersection (also see magnified transmittance map in Supplementary Fig. S4). The exact formation of this unique Fano resonance is schematically illustrated in Fig. 3. The plane wave excitation with the incident electric field (*E*) polarized as indicated by the blue arrow induces the parallel ED (*p*) and EQ (*Q*_*e*_) and perpendicular MQ moments (*Q*_m_) inside the resonators (Fig. 3a, b). The excited resonances interfere with the coupling strength *κ*, forming the typical asymmetric resonance profile in the resulting transmittance spectrum (Fig. 3c), which, in turn, produces a strong TH emission (Fig. 3d). The width and spectral position of the individual resonances and thus the Fano-resonance can be easily tuned by varying the elliptical diameters *d*_x_ and *d*_y_. In the special case of highly fine-tuned resonances, the sharp asymmetric resonance feature in the transmission spectrum disappears and a Lorentz resonance profile is formed^25,43^.

We follow our analysis by investigating the nonlinear response of the metasurface in the considered range of parameters. The calculated intensity of the THG signal together with the superimposed contour isolines of the modes from the transmittance map revealed by the linear response are shown in Fig. 2c. Notably, despite the considered absorption losses in the resonator (also see the a-Si dispersion curve in Supplementary Fig. S2), the TH enhancement features found in this map follow strictly the MQ/Fano mode path, indicating their dominant impact on the generated high harmonic radiation. Furthermore, a quick glance at the electric field amplitude maps at the third harmonic, presented in Fig. 2e, shows that the enhanced TH emission is supported by the relatively outer location of the THG hotspots compared to the more inner located hotspots at the other resonances. The TH intensity spans twelve orders of magnitude with the highest magnitude being found near the intersection of the MQ/Fano with ED and EQ modes in the region, where the Super-Fano mode exists. The maximal THG is found for elliptical diameters of *d*_*x*_ ~ 740 nm and *d*_*y*_ ~ 550 nm. In a simplified picture, the ultra-narrow high-Q linear resonance is connected to locally enhanced fields, which lead to a THG enhancement due to the polynomial exponent of the nonlinear process.

### Experimental analysis

**Fig. 4:**
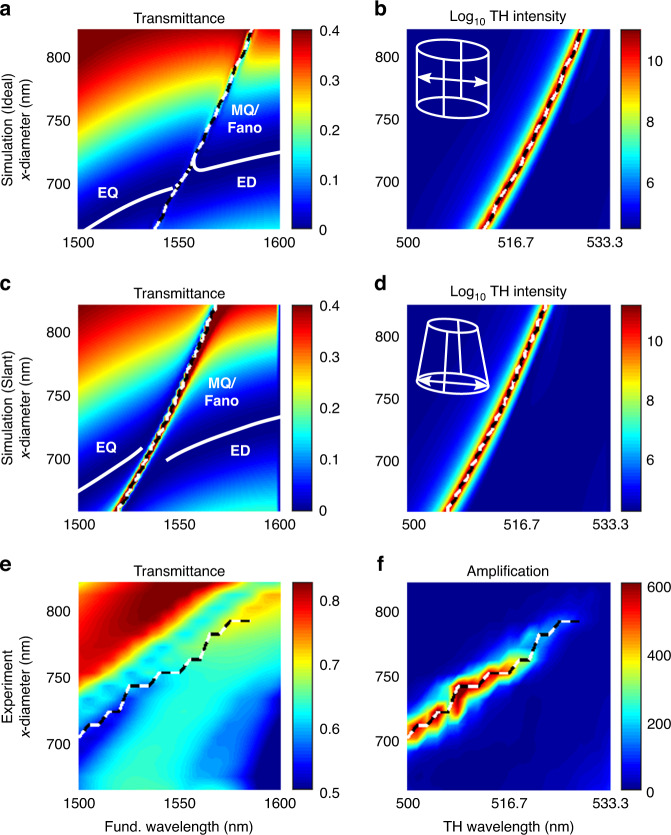
Transmittance and THG intensity maps. **a** Calculated spectral transmittance and (**b**) THG intensity maps of the metasurface as a function of the nanodisk elliptical diameter *d*_*x*_ with perfectly vertical sidewalls. The dashed curve in both maps marks the linear MQ/Fano resonance, which coincides with the maximum of THG in map (**b**). **c**, **d** The same as panel (**a**, **b**) but for nanodisks with 1° slanted sidewalls. **e** Corresponding experimental spectral transmittance and (**f**) TH amplification maps of the fabricated metasurface

To verify our theoretical findings of ultrahigh THG, we fabricated and analyzed 100 × 100 μm^2^ patches of a-Si cylinders on glass substrate using the methods described in the sections ‘Fabrication’ and ‘Experimental measurement methods’. As seen in the SEM image of the fabricated structure in Fig. 1b, the side walls of the Si nanoantennas are not perfectly vertical, but slightly slanted. This effect is often unavoidable and frequently seen in similar dielectric structures. Therefore, we numerically investigated the impact of slanted sidewalls on THG. For this, we assumed a sidewall angle of 1° and compared the results for both, the linear transmission as well as the nonlinear THG intensity. The results are shown in Fig. 4a–d. Note that in this figure, the results are plotted for a fixed *y*-diameter of *d*_*y*_ = 550 nm for various *d*_*x*_ over the fundamental/THG wavelength. From these data, it can immediately be seen that already a small sidewall angle leads to a significant blue-shift of the observed resonance. A more careful analysis of the individual spectra also reveals a broadening of the Fano resonance itself for the resonators with slanted sidewalls. Consequently, a similar effect is expected in the experimental results. Nevertheless, for both calculations, a strong enhancement of the THG signal is expected, as the THG intensity is predicted to be several orders of magnitude higher than for the non-resonant case.

Compared to the narrow resonance stripe in the theoretically calculated transmittance maps in Fig. 4a, c, the width of the resonance stripe in the experimentally measured transmittance map in Fig. 4e is much wider. This broadening is caused by losses not accounted for in the simulation model. Some of the contributions to these losses are, e.g., the two-photon absorption (TPA), manufacturing inaccuracies such as sidewall roughness and the numerical aperture, i.e., the large acceptance angle of the used Cassegrain objective in the FTIR setup. Moreover, the inserted black and white dashed line in the transmittance map is not exactly aligned with the resonance feature, like in the transmittance map in Fig. 4a, and is slightly tilted. Comparing the experimental results for the nonlinear THG intensity in Fig. 4f with the numerical simulations in Fig. 4b, d, a qualitative agreement is found. However, also here, a stronger dependence on the *x*-diameter *d*_*x*_ is found than in the numerical simulations. Another interesting observation in the experimental result for the THG intensity is the fact that the THG amplification vanishes for larger values of *d*_*x*_ and *λ*. But this is not the case in the numerical results, where the resonant THG enhancement is found to be strong in the entire range of *d*_*x*_ values. A possible explanation for this might be the large excitation wavelength range used in the experiments, which causes the pulse profile and, therefore, the peak power intensities to change with varying wavelength even if the mean power is kept constant.

Finally, it should be pointed out again that the change of the resonator *x*-diameter theoretically leads only to a wavelength shift of the resonance and not to a change of the intensity over a large range. This behavior can also be expected from the maps in Fig. 2b, c, where a nearly horizontal resonance feature occurs without a large change in the *y*-diameter around the optimum labeled with Super-Fano in the parameter space map. In contrast, changing the *y*-diameter alone leads to a significant decrease in the generated THG, see the corresponding maps in Fig. 2 and Supplementary Fig. S5. Therefore, the *x*-diameter represents an excellent parameter for fine-tuning the enhancement of THG for a given wavelength. Moreover, the reduction in THG caused by the change in *y*-diameter can be compensated by an additional change in x-diameter. The corresponding maps are included in Supplementary Fig. S6.

**Fig. 5:**
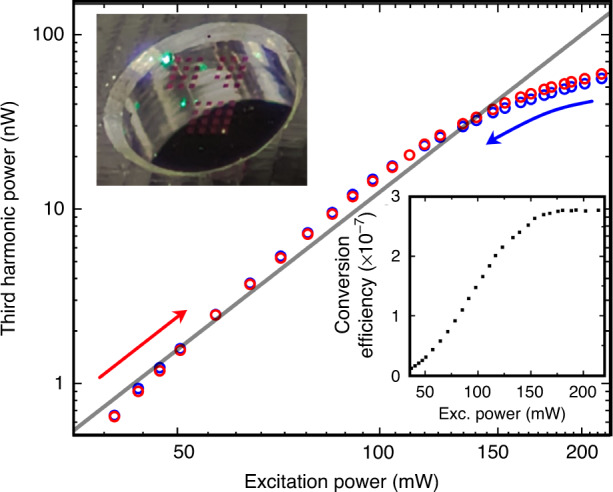
The third harmonic power as a function of the pump power (log-log scale). Red and blue circles correspond to the gradually increasing or decreasing pump power, respectively. The gray line represents the cubic law. The bottom right inset shows the achieved conversion efficiency as a function of the pump power. The upper left inset presents a photograph showing third harmonic green light emission from the sample for the pump wavelength of 1560 nm

In the final step of the investigation, we determine the maximum possible TH conversion efficiency of the proposed optimal metasurface. Therefore, the power of the pump beam *P*_in_ (*ω*) and the radiated TH signal *P*_out_ (3*ω*) are measured as described in the experimental measurement methods ‘Experimental measurement methods’ and shown in Fig. 5. The inset shows the achieved conversion efficiency dependent on the excitation power. A deviation in the cubic law can be observed for high peak intensities above approximately 150 mW in contrast to the as-grown thin film (see Supplementary Fig. S8) indicating that the undepleted pump approximation is not applicable anymore. This saturation can also be observed in the conversion efficiency, where its value maximizes with *η*_max_ ≈ 2.8 × 10^−7^ at a peak power intensity of approx. 1.2 GW cm^−2^ (estimated from the excitation power assuming 80 fs long pulses at 80 MHz repetition rate). Related approaches to the enhancement of THG have been examined in refs. ^44,18^, where conversion efficiencies of 1 × 10^−7^ at 5.5 GW cm^−2^ and 1.2 × 10^−6^ at 3.2 GW cm^−2^ were achieved, respectively.

In comparison, we reach a similar conversion efficiency with a much lower peak power intensity. However, it should be noticed that due to the very high Q-factor, our developed Super-Fano THG process is an ultra-narrow nonlinear process, which can consequently transfer only a very small part of a general broad or ultra short input pulse into the third harmonic. Accordingly, a temporally longer pulse at the same power should significantly increase the achievable conversion efficiency. Apart from this, the designed structure is still very attractive for various applications where, for example, very little space is available, and is also capable of producing a strong light visible to the naked eye in less than a wavelength, as shown in the small photo in Fig. 5. Moreover, maximum amplification factors of up to ~900 are reached compared to an unpatterned a-Si thin film of same thickness. Polarization dependent measurements show the expected cos^6^ dependency of the THG intensity, as can be seen in Supplementary Fig. S7, proving the sole attribution of the Fano-resonant structure on the enhancement in THG.

## Discussion

In summary, it is shown that a periodically arranged array of elliptical nanoresonators made of amorphous silicon exhibits strong light-matter interaction due to Fano resonances. Theoretical calculations reveal that these Fano resonances are the result of coupling between a high-quality magnetic quadrupole and a broad electric dipole. As predicted by theory, the strong light-matter interaction can be used to extremely enhance the generation of third harmonic light, especially if additionally interacting with the electric quadrupole mode as further constituent leading to the formation of ultra-narrow features, which we call ‘Super-Fano’. Amplification factors of up to ~900 compared to an unpatterned a-Si thin film of same thickness are achieved. A maximum conversion efficiency of *η*_max_ ≈ 2.8 × 10^−7^ at a peak power intensity of approx. 1.2 GW cm^−2^ is measured.

## Materials and methods

### Fabrication

To achieve the desired enhancement of THG by Fano resonances in amorphous silicon metasurfaces the design depicted in Fig. 1a is proposed. Our fabrication process includes the following steps. First, 590nm amorphous silicon (a-Si) is deposited on a glass substrate using a plasma-enhanced chemical vapor deposition (PECVD) process with 400 sccm of SiH_4_/Ar mix (2%/98%) at 300 °C, 1 torr chamber pressure and 10 W rf power. Electron beam lithography (EBL) is used to define a mask for the subsequent pattern transfer. The adhesion promoter AR 300-80 is spin-coated with 400 rpm for 40 s onto the a-Si thin film and baked at 90 °C for 2 min. Then, the positive electron beam resist AR-P 679.03 (PMMA 950k) is spin-coated with 4000 rpm for 40 s and baked at 180 °C for 10 min. In order to mitigate charging effects during lithography caused by the insulating property of the glass substrate, a thin layer of the conductive protective coating Electra 92 AR-PC 5090 is spin-coated with 5500 rpm for 40 s and baked at 90 °C for 2 min. EBL is performed at 20 kV with an area dose of 220 μC cm^−2^. The sample is developed in subsequent baths of de-ionized water for 60 s and the developer AR 600-56 for 75 s to remove the Electra 92. Isopropyl alcohol is used to stop the development process. A lift-off procedure is then employed to fabricate a metallic hard mask. For this, electron beam evaporation is used to deposit a 20 nm thin chromium film onto the developed sample. For the lift-off process the sample is soaked in 80 °C warm acetone for 1 h before an ultrasonic cleaner is used to remove all resist and unwanted chromium from the sample. The ramaining structured chromium mask is needed for the pattern transfer into the a-Si thin film. This pattern transfer is accomplished by anisotropic inductively coupled plasma (ICP) reactive ion etching (RIE) in a gas mixture of 45 sccm C_4_F_8_ and 18 sccm SF_6_ with an ICP power of 900 W and rf power of 41W. Helium backside cooling is used with a helium flow of 7 sccm. The sample is etched for 2 min 5 s to expose the glass substrate in regions where no chromium hard mask is present. Finally, the strucutre is completed by wet chemical etching of the remaining chromium with a 1: 1 dilution of the chromium etchant TechniEtch Cr01 and de-ionized water for 15 min. With this technique 100 × 100 μm^2^ antenna fields consisting of elliptical a-Si nanoantennas arranged in a square lattice are fabricated, as sketched in Fig. 1a. The lattice parameter *p* = 916.7 nm is kept constant for every antenna field and the height of the antennas *h* = 590 nm is given by the thickness of the a-Si film. The diameters in *x*- and *y*-direction of the antennas are varied between different antenna fields. Figure 1b shows a scanning electron microscope (SEM) image of such an antenna.

### Experimental measurement methods

Linear transmittance measurements are performed by FTIR-spectroscopy. Here, an adjustable rectangular aperture is used to limit the measured area to a single antenna field. As reference, the bare exposed SiO_2_ substrate is measured which later allows to deduce the effect of the Si nanoantenna field on the transmittance. Two customized optical setups are used for nonlinear measurements. One is shown schematically in Fig. S1 of the Supplementary Material. The used 200 fs-pulsed Ti-sapphire laser pumping an optical parametric oscillator (OPO) allows for wavelength-dependent analysis of the fabricated antenna fields. A half-wave plate and a following polarizing beam splitter (PBS) are used for controlling the laser power. For all measurements the average laser power is adjusted to *P* = 75 mW using an optical power meter (OPM), unless otherwise stated. A second half-wave plate is used to rotate the polarization of the laser light and thus change the excitation polarization. The laser beam is focused onto an antenna field from the backside of the sample by a lens with focal length f = 50 mm, resulting in a laser spot with a diameter of about 50 μm. The sample can be moved in two directions allowing for accurate positioning of the laser beam onto an antenna field in combination with the used camera. This guarantees that only the nonlinear signal from a single antenna field is collected by an objective with 20× magnification and a numerical aperture of NA = 0.5. A long-pass dichroic mirror is used to filter out the excitation wavelength, so only the third harmonic signal is collected by the spectrometer. An unpatterned a-Si thin film is used as reference to directly calculate the amplification of the THG signal resulting from the nanoantenna array without consideration of the nonlinear active volume.

The second nonlinear setup uses a similar measurement geometry and is employed for power dependent measurements of the investigated THG process (not shown here). It uses a fiber laser emitting monochromatic light with a wavelength of 1560 nm allowing for higher and more stable excitation powers. The power can be adjusted in software making the power control via half-wave plate and PBS obsolete. Any THG signal generated by optical components like the half-wave plate for polarization control in front of the sample is filtered by a long-pass filter. The excitation laser is filtered by a band-pass filter after the sample, to prevent distortion in the measured THG power by an OPM. With this, the THG conversion efficiency *η* = *P*_out_ (3*ω*)/*P*_in_ (*ω*) is readily obtained, where *P*_in_ (*ω*) is the power of the pump beam and *P*_out_ (3*ω*) the radiated TH signal power^7^.

### Theoretical methods

For the theoretical calculations of the linear and nonlinear response from the metasurfaces, we use a commercial tool based on the finite element method (FEM)^45^. The general simulation model, a unit cell of the infinitely periodic surface, consists of a single elliptical cylinder made of amorphous silicon (a-Si) placed on a glass (silicon dioxide, SiO_2_) substrate block, as shown in Fig. 2a. The periodicity of the structure is modeled by introducing periodic boundary conditions on the sidewalls of the unit cell in the *x*- and *y*-directions, while open boundary conditions with perfectly matched layers (PML) are added above and below the resonator in *z*-direction. The unit cell model is excited from the air-side by a wave source port closely below the PML, which emits a vertically (*z*-direction) propagating plane wave linearly polarized in the *x*-direction at the fundamental frequency/wavelength. To account for the dispersive dielectric function of the amorphous silicon, we use experimental results obtained employing spectroscopic ellipsometry. The corresponding model parameter and dispersion curves are provided in Supplementary Table S1 and Fig. S2. For the dielectric function of the silicon dioxide, the experimentally measured values from the database by *Malitson*^46^ are used. Since silicon is a centrosymmetric material, there are no contributions to second-order nonlinear processes from the bulk crystal. Thus, third-order nonlinear processes, such as THG, are the lowest order nonlinear processes occurring in silicon. In simulations, the third-order nonlinearity is taken into account via the nonlinear polarization term ${{{\mathbf{P}}}}_{{{{\mathbf{NL}}}}}^{\left( 3 \right)}(3\omega ) = \varepsilon _0\chi ^{\left( 3 \right)}({{{\mathbf{E}}}}\left( \omega \right) \cdot {{{\mathbf{E}}}}(\omega )){{{\mathbf{E}}}}(\omega )$ with the constant value of 2.45 × 10^−19^m^2^V^−2^ for the nonlinear susceptibility *χ*^(3)^ (ref. ^47^.). The back coupling of the generated third harmonic on the fundamental signal is assumed to be so small that it can be neglected. Due to this, the simulations are performed in two calculation steps, starting with the plane wave excitation at the fundamental frequency followed by an external current excitation generated by the nonlinear polarization with ${{{\mathbf{J}}}}_{{{{\mathrm{ext}}}}} = j3\omega {{{\mathbf{P}}}}_{{{{\mathbf{NL}}}}}^{\left( 3 \right)}(3\omega )$ at the third harmonic within the silicon resonator. The corresponding far-field quantities at the fundamental frequency and third harmonic are extracted with a near-field to far-field transformation (NFFFT) for periodic structures^48^, which resembles the integral for the two-dimensional complex Fourier series. However, only the emitted zeroth diffraction order (*k*_*x*_ = *k*_*y*_ = 0) in transmission or reflection direction is considered in this work.

## Supplementary information

Supplementary materials

## References

[1] Boyd, R. W. *Nonlinear Optics*. 3rd edn. (Waltham: Elsevier, 2008).

[2] Blount EI, Klauder JR (1969). Recovery of laser intensity from correlation data. J. Appl. Phys..

[3] Diels JCM (1985). Control and measurement of ultrashort pulse shapes (in amplitude and phase) with femtosecond accuracy. Appl. Opt..

[4] Ramos-Ortiz G (2004). Third-order optical autocorrelator for time-domain operationat telecommunication wavelengths. Appl. Phys. Lett..

[5] Trebino R (1997). Measuring ultrashort laser pulses in the time-frequency domain using frequency-resolved optical gating. Rev. Sci. Instrum..

[6] Johnson AS (2020). Measurement of 10 fs pulses across the entire Visible to Near-Infrared Spectral Range. Sci. Rep..

[7] Pimputkar S (2009). Prospects for LED lighting. Nat. Photonics.

[8] Koenderink AF, Alù A, Polman A (2015). Nanophotonics: shrinking light-based technology. Science.

[9] Lozano G (2016). Metallic nanostructures for efficient LED lighting. Light Sci. Appl..

[10] Zheludev NI, Kivshar YS (2012). From metamaterials to metadevices. Nat. Mater..

[11] Chen SQ (2018). Phase manipulation of electromagnetic waves with metasurfaces and its applications in nanophotonics. Adv. Opt. Mater..

[12] Pertsch T, Kivshar Y (2020). Nonlinear optics with resonant metasurfaces. MRS Bull..

[13] Solntsev AS, Agarwal GS, Kivshar YS (2021). Metasurfaces for quantum photonics. Nat. Photonics.

[14] Krasnok A, Tymchenko M, Alù A (2018). Nonlinear metasurfaces: a paradigm shift in nonlinear optics. Mater. Today.

[15] Myroshnychenko V (2008). Modelling the optical response of gold nanoparticles. Chem. Soc. Rev..

[16] Kauranen M, Zayats AV (2012). Nonlinear plasmonics. Nat. Photonics.

[17] Linden S (2012). Collective effects in second-harmonic generation from split-ring-resonator arrays. Phys. Rev. Lett..

[18] Yang YM (2015). Nonlinear fano-resonant dielectric metasurfaces. Nano Lett..

[19] Shorokhov AS (2016). Multifold enhancement of third-harmonic generation in dielectric nanoparticles driven by magnetic fano resonances. Nano Lett..

[20] Cui CC (2018). Multiple fano resonances in symmetry-breaking silicon metasurface for manipulating light emission. ACS Photonics.

[21] Grinblat G (2017). Efficient third harmonic generation and nonlinear subwavelength imaging at a higher-order anapole mode in a single germanium nanodisk. ACS Nano.

[22] Li Y (2020). Optical anapole mode in nanostructured lithium niobate for enhancing second harmonic generation. Nanophotonics.

[23] Carletti L (2018). Giant nonlinear response at the nanoscale driven by bound states in the continuum. Phys. Rev. Lett..

[24] Koshelev K (2020). Subwavelength dielectric resonators for nonlinear nanophotonics. Science.

[25] Melik-Gaykazyan E (2021). From fano to quasi-bic resonances in individual dielectric nanoantennas. Nano Lett..

[26] Xiao SY (2022). Robust enhancement of high-harmonic generation from all-dielectric metasurfaces enabled by polarization-insensitive bound states in the continuum. Opt. Express.

[27] Li GX, Zhang S, Zentgraf T (2017). Nonlinear photonic metasurfaces. Nat. Rev. Mater..

[28] Chen SM (2018). Controlling the phase of optical nonlinearity with plasmonic metasurfaces. Nanophotonics.

[29] Grinblat G (2021). Nonlinear dielectric nanoantennas and metasurfaces: frequency conversion and wavefront control. ACS Photonics.

[30] Liu S (2016). Resonantly enhanced second-harmonic generation using III–V semiconductor all-dielectric metasurfaces. Nano Lett..

[31] Wang C (2017). Metasurface-assisted phase-matching-free second harmonic generation in lithium niobate waveguides. Nat. Commun..

[32] Golla C, Weber N, Meier C (2019). Zinc oxide based dielectric nanoantennas for efficient nonlinear frequency conversion. J. Appl. Phys..

[33] Tong WY (2016). Enhanced third harmonic generation in a silicon metasurface using trapped mode. Opt. Express.

[34] Gao YS (2018). Nonlinear holographic all-dielectric metasurfaces. Nano Lett..

[35] Schlickriede C (2020). Nonlinear imaging with all-dielectric metasurfaces. Nano Lett..

[36] Kruk SS (2022). Asymmetric parametric generation of images with nonlinear dielectric metasurfaces. Nat. Photonics.

[37] Reineke B (2019). Silicon metasurfaces for third harmonic geometric phase manipulation and multiplexed holography. Nano Lett..

[38] Liu BY (2020). Nonlinear wavefront control by geometric-phase dielectric metasurfaces: influence of mode field and rotational symmetry. Adv. Opt. Mater..

[39] Wang L (2018). Nonlinear wavefront control with all-dielectric metasurfaces. Nano Lett..

[40] Santiago-Cruz T (2021). Photon pairs from resonant metasurfaces. Nano Lett..

[41] Santiago-Cruz T (2022). Resonant metasurfaces for generating complex quantum states. Science.

[42] Hähnel, D., Förstner, J. & Myroshnychenko, V. Efficient nonlinear wavefront shaping by dielectric metasurfaces. Preprint at 10.48550/arxiv.2209.15384 (2022).

[43] Liu YN (2022). Full control of fano spectral profile with gst-based metamaterial. ACS Photonics.

[44] Shcherbakov MR (2014). Enhanced third-harmonic generation in silicon nanoparticles driven by magnetic response. Nano Lett..

[45] COMSOL Multiphysics. www.comsol.com, COMSOL AB, 2020.

[46] Malitson IH (1965). Interspecimen comparison of the refractive index of fused silica. J. Opt. Soc. Am..

[47] Hon NK, Soref R, Jalali B (2011). The third-order nonlinear optical coefficients of Si, Ge, and Si_1−*x*_Ge_*x*_ in the midwave and longwave infrared. J. Appl. Phys..

[48] Taflove, A. & Hagness, S. C. in *Computational Electrodynamics: The Finite-Difference Time-Domain Method*. 3rd edn, (Boston: Artech House, 2005).

