# Supplementary material for: A multi-mode super-fano mechanism for enhanced third harmonic generation in silicon metasurfaces
Source: Light Sci Appl. 2023 Apr 21;12:97. doi: 10.1038/s41377-023-01134-1 (PMC10119293; doi:10.1038/s41377-023-01134-1)
Supplement: Supplementary file 1 — Supplementary materials [file 41377_2023_1134_MOESM1_ESM.pdf]

Supplementary Materials for:

A Multi-Mode Super-Fano Mechanism for Enhanced Third Harmonic Generation in Silicon Metasurfaces

David Hähnel [1,*], Christian Golla [2,†], Maximilian Albert [2], Thomas Zentgraf [2], Viktor Myroshnychenko [1], Jens Förstner [1], and Cedrik Meier [2]

[1] *Theoretical Electrical Engineering & CeOPP, Paderborn University, 33098 Paderborn, Germany.*
[2] *Physics Department & CeOPP, Paderborn University, 33098 Paderborn, Germany.*

* hdavid@mail.uni-paderborn.de
† christian.golla@uni-paderborn.de

Schematic of the setup for nonlinear optical measurements

A schematic of the optical setup used for wavelength-dependent nonlinear optical measurements is shown in Fig. S1.

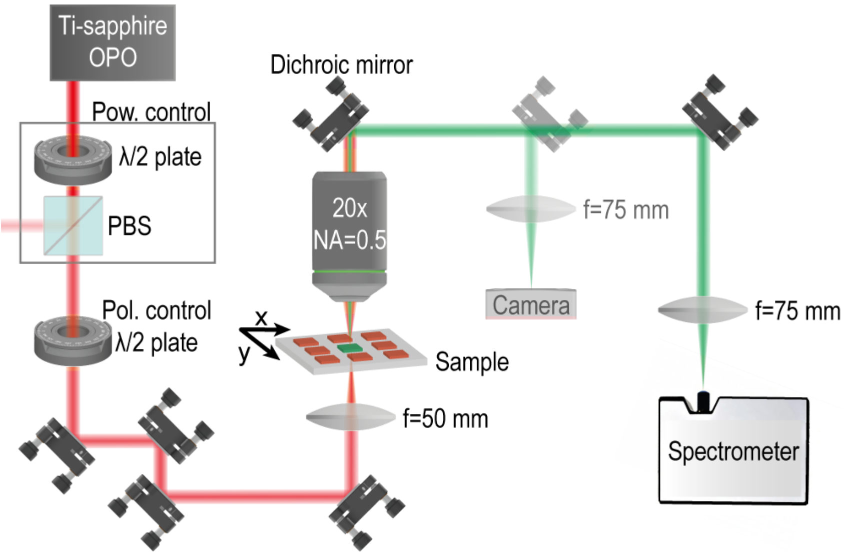

Figure S1: Schematic of the used optical setup for wavelength dependent nonlinear measurements.

Dielectric function of amorphous silicon

The dielectric function of the amorphous silicon films have been measured using spectroscopic ellipsometry. As a light source, a xenon arc lamp was used in combination with a grating monochromator. The light was detected using a silicon/(In,Ga)As stack photodiode, so that measurements in the range from 400 nm–1700 nm were feasible. The glass substrate was modelled using a Cauchy model following

$$n(\lambda) = A_n + \frac{B_n}{\lambda^2} + \frac{C_n}{\lambda^4}$$

where λ is given in µm.

For the a-Si thin film a Cody-Lorentz model was employed. The relevant equations can, e. g. be found in Di et al. [1]. To further enhance the model accuracy, the surface roughness of the a-Si film was modelled using effective medium approximation with 50 % volume inclusion of void, here air. The fit-parameters can been seen in Tab. S1.

Glass - Cauchy model

A_n	B_n	C_n
1.4316	0.01571	-0.00181

Amorphous silicon (a-Si) - Cody-Lorentz model

$E_{1,\text{offset}}$	A	E_{on}	Γ	E_g	E_p	E_t	E_μ
-0.53402	88.767	4.3407	2.9351	1.6981	1.0951	0.089385	1

Table S1: Fit-parameters obtained in the spectroscopic ellipsometry measurements.

The resulting values for the refractive index n and the extinction coefficient k are shown in Fig. S2.

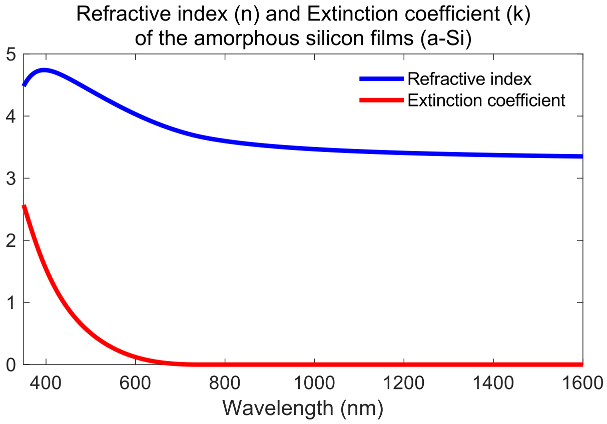

Figure S2: Experimentally measured refractive index and extinction coefficient of the a-Si films from spectroscopic ellipsometry.

Super-Fano evolution spectra

The evolution of the transmittance spectra toward the super-Fano resonance for six selected geometry parameters along the MQ/Fano curve in Fig. 2 of the paper is given in Fig. S3a. The minima in the spectra are caused by the absorption of the occuring modes. In the first spectrum, far from the super Fano conditions (dark blue curve in the upper part of the diagram), the modes are most clearly separated and distinct. One can clearly see the typical asymmetric Fano line shape of the MQ/Fano resonance resulting of the interaction of the narrow MQ mode with the very broad nearby ED mode. This asymmetric line shape steepens when moving towards larger x-diameters, and most notable the EQ mode shifts towards the MQ/Fano resonance leading to increasing interaction between them. Finally, for the last spectrum (light blue blue curve in the bottom part of the diagram), this leads to the Super-Fano formation with an ultra-narrow high-Q dispersive shape. For a better verification of the above described convergence process of the three resonance modes - ED, MQ and EQ - in the wavelength spectra with the presented convergence of the resonances in the transmittance map in Fig. 2 of the paper, a more

detailed visualization of the corresponding paths is included in Fig. S4.

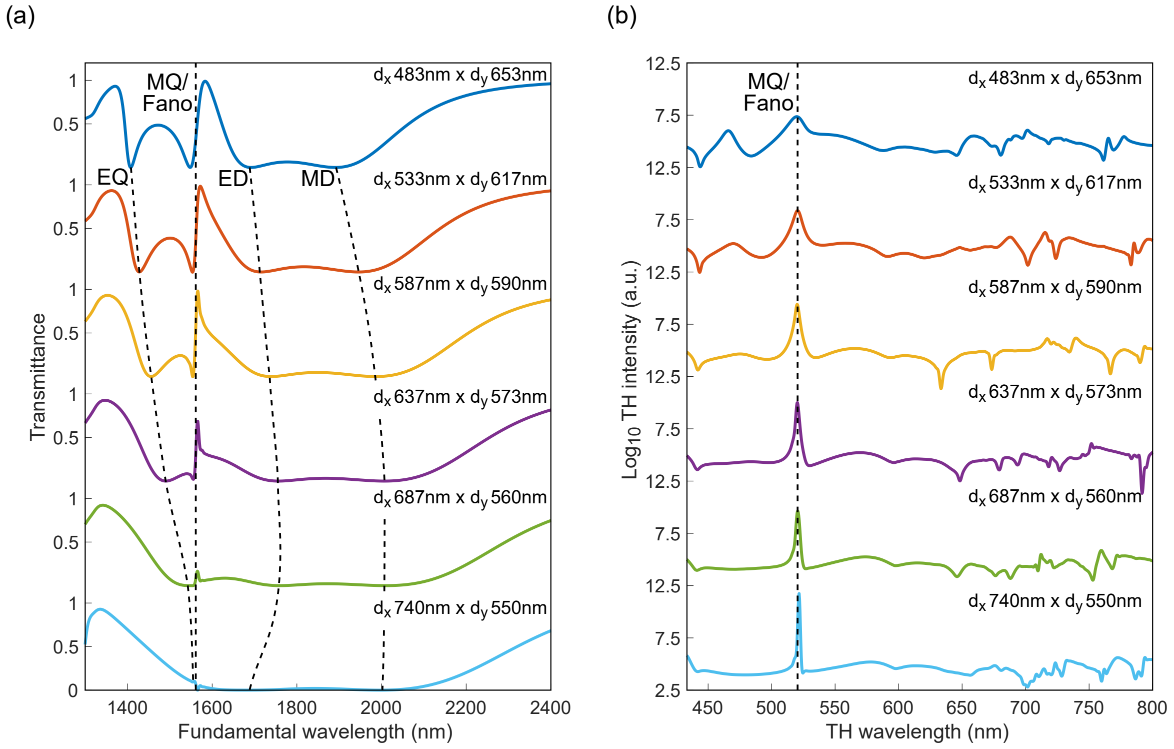

Figure S3: (a) Transmittance spectra for six selected geometrical parameters along the MQ/Fano curve in Fig. 2 of the paper. The minima in the spectra corresponds to the absorption by the modes (marked as EQ, MQ/Fano, ED, MD). The evolution of the mode frequencies for changing geometrical parameters is visually supported by the dashed curves. Note the merging of the EQ with the MQ/Fano mode in the lowest curve representing the Super-Fano formation ($d_x = 740\,\mathrm{nm}$, $d_y = 550\,\mathrm{nm}$). (b) THG spectra for the same geometries as in (a). Note the high THG signal at the spectral position of the MQ/Fano resonance, which peaks in the regime of Super-Fano formation.

In the THG spectra in Fig. S3b one can observe strong enhancement of the THG at the spectral position of the MQ/Fano mode, which becomes extreme in the region of Super-Fano formation (lowest curve).

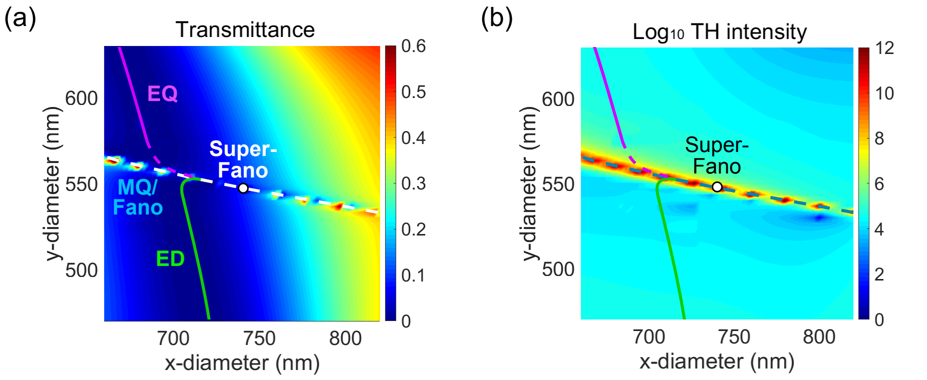

Figure S4: (a) Magnified transmittance and (b) decadic logarithm TH intensity map of the maps in Fig. 2 around the Super-Fano resonance point at $d_x = 740\,\mathrm{nm}$ and $d_y = 550\,\mathrm{nm}$. The corresponding ED, MQ, EQ and the Super-Fano resonance are marked in the same way as in Fig. 2. To highlight the asymmetric resonance feature of the Fano resonance (dashed line) in the transmittance map (a), the color bar is confined to the value of 0.6.

Dependence of the Resonance on d_y

While the numerically predicted THG intensity does not strongly depend on d_x, it is significantly affected by d_y. Fig. S5 shows the wavelength dependence of the simulated and experimentally measured THG intensity for different d_y values at $d_x = 740\,\mathrm{nm}$.

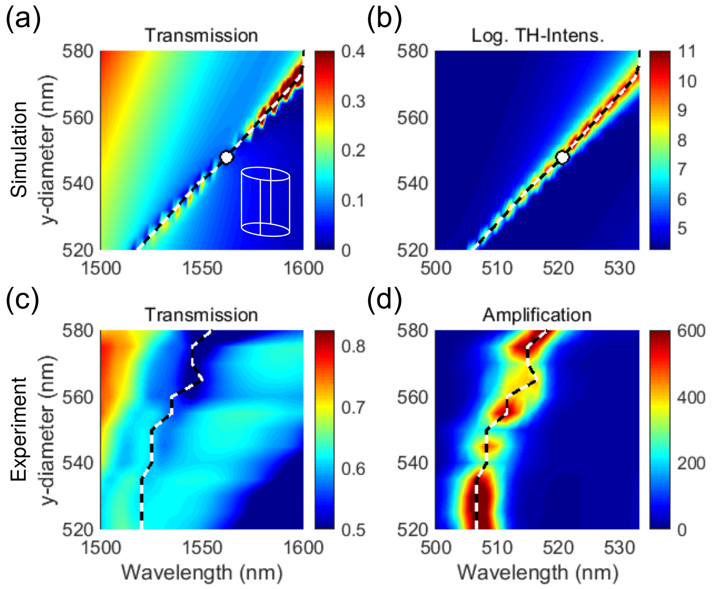

Figure S5: Wavelength dependent results for the linear transmission and the nonlinear THG intensity for a fixed x-diameter $d_x = 740\,\mathrm{nm}$.

Experimental demonstration of maximum THG amplification

In order to find the highest experimental values for the THG amplification, the geometrical parameters (d_x, d_y) were varied in 1 nm steps. The results are shown in Fig. S6. The maximum measured THG amplification was 862.

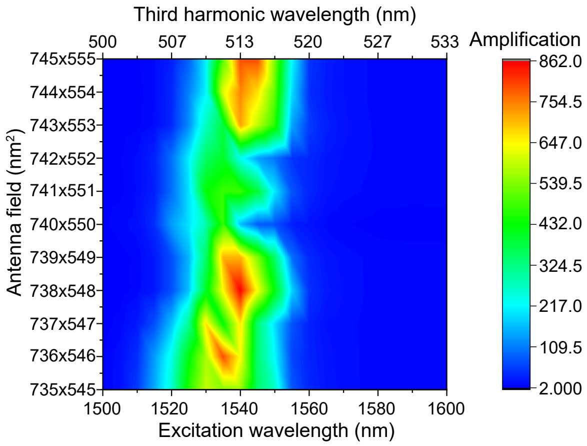

Figure S6: Experimental values for the THG amplification in a small range of (d_x, d_y).

Polarization dependency

Polarization dependent measurements were performed on an antenna field, by rotating the polarization angle of the laser light. The most THG is generated at the polarization angles 90° and 270° where the electric field vector is parallel to the longer elliptical axis d_x (see Fig. S7). As expected with $E_x = E\cos(\alpha)$ and $I_{\mathrm{THG}} \propto E_{\mathrm{THG}}^2 \propto E\cos(\alpha)^6$, the polarization dependent measurement shows a $\cos^6$ dependency. This further proves that the strong amplification of THG is attributed to the Fano-resonant character of the antenna fields.

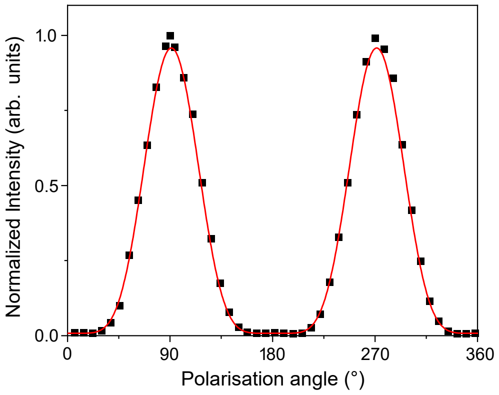

Figure S7: Experimental results for the polarization dependency of the THG intensity.

Power dependency of the as-grown thin film

Power dependent measurements were performed on the as-grown silicon thin film and are shown in Fig. S8. The figure shows the area under the THG peak measured with a spectrometer in dependence of the laser power. The measurements on the antenna field show the deviation in the cubic law at high excitation powers (around 150 mW) as already shown in the paper. The as-grown sample does not exhibit this behavior. We explain this with the absence of any dielectric modes in the as-grown thin film, which result in no enhancement of the electric field in contrast to the nanoresonators. Due to the Fano resonance an its accompanied high electric field enhancement inside the nanoresonators the non-applicability of the undepleted pump approximation is reached faster/with lower powers than in the thin film.

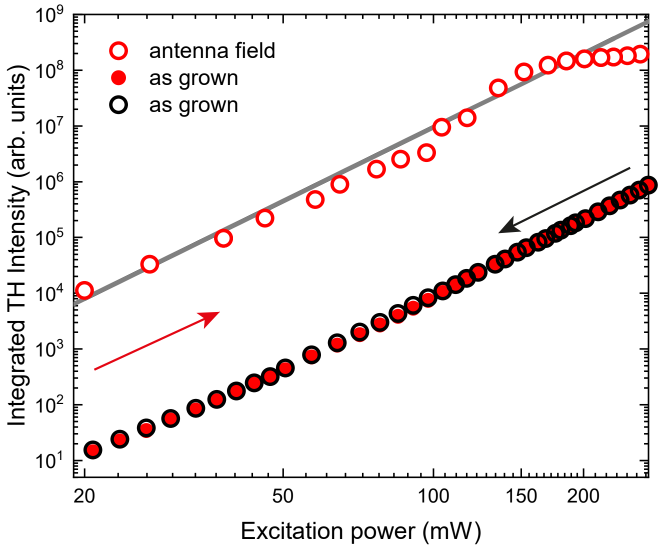

Figure S8: Experimental results for the power dependency of the THG intensity of the as-grown silicon thin film compared to an antenna field.

References

[1] Di, M. *et al.* Comparison of methods to determine bandgaps of ultrathin HfO_2 films using spectroscopic ellipsometry. J. Vac. Sci. Technol. A **29**, 041001 (2011).
